# Gliomatosis cerebri mimicking acute viral encephalitis and with malignant transformation of partial lesions: A case report

**DOI:** 10.3892/etm.2014.1807

**Published:** 2014-06-24

**Authors:** PEIXIN SUN, HAOZHE PIAO, XU GUO, ZHENGRONG WANG, RUI SUI, YE ZHANG, BING YAO, YI CHEN

**Affiliations:** 1 Department of Neurosurgery, Liaoning Cancer Hospital and Institute, Shenyang, Liaoning 110042, P.R. China; 2 Department of Thoracic Surgery, Liaoning Cancer Hospital and Institute, Shenyang, Liaoning 110042, P.R. China

**Keywords:** gliomatosis cerebri, viral encephalitis, malignant transformation

## Abstract

Gliomatosis cerebri (GC) is a rare glial neoplasm, characterized by extensive diffuse brain infiltration and relative preservation of the underlying architecture. In the present case report, a patient with type 2 GC, which mimicked the clinicoradiological course of acute viral encephalitis, is presented. A 56-year-old male presented with fever, dizziness, headache and numbness in the right extremities three days prior to admission to hospital. The cerebrospinal fluid (CSF) showed mild pleocytosis. Brain magnetic resonance imaging (MRI) revealed hyperintensity on fluid-attenuated inversion recovery images in the left frontal, temporal, insular lobes and in the left thalamus. No signal enhancement was observed following gadolinium administration. The patient was diagnosed with acute viral encephalitis of unknown cause and received a 10-day course of acyclovir, intravenously. At the follow-up three months later, the patient had personality changes and memory deterioration. The results from the follow-up MRI revealed no remarkable changes. At the follow-up six months after presentation, the patient had expressive aphasia and severe headaches. Subsequently, the patient had two tonic-clonic seizure onsets. The results from the MRI showed an increase in lesion size, more edema around the lesion and irregular enhancement in the left frontal lobe. However, the lesions in the left temporal and insular lobes and in the left thalamus were nearly unchanged. Magnetic resonance spectroscopy (MRS) showed elevated choline (Cho)/creatine (Cr) and Cho/*N*-acetylaspartate (NAA) ratios, as well as decreased NAA/Cr ratios. Surgery was performed and the neuropathological diagnosis of WHO grade III astrocytoma was confirmed. Thus, it is important to pay attention to the differential diagnoses of GC and acute viral encephalitis in patients who have widespread MRI lesions. A brain biopsy is recommended for a diagnosis in this case.

## Introduction

Encephalitis is an unusual manifestation of human viral infection; only a few patients who have acquired systemic viral infections develop symptomatic infection of the central nervous system. Acute viral encephalitis is characterized by a triad of fever, headache and an altered level of consciousness (1). Other clinical characteristics include behavior and speech disturbances, disorientation, hemiparesis and seizures. The most common cause of sporadic encephalitis is herpes simplex virus (HSV) type I, which may be diagnosed by methods including serological testing for IgM antibodies, polymerase chain reaction (PCR) analysis to search for HSV DNA or brain biopsy (2). Computed tomography (CT) scans and magnetic resonance imaging (MRI) scans may also provide useful information.

An alternative situation, in which a gliomatosis cerebri (GC) presents as an acute encephalitic illness, has rarely been reported. To the best of our knowledge, only one case has previously been reported (3). GC is a rare tumor of the central nervous system, and the World Health Organization (WHO) criteria define GC as a diffusely infiltrative glioma that involves at least three cerebral lobes (4). In the present study, a patient with GC, which mimicked the clinicoradiological course of acute viral encephalitis, is presented.

## Case report

**Figure 1:**
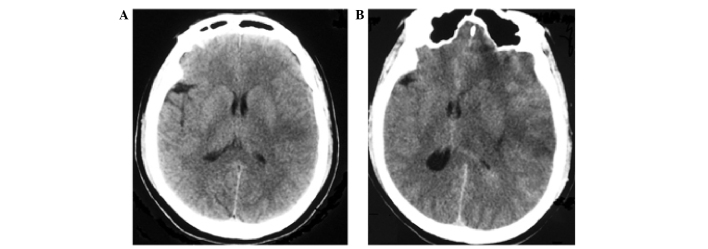
CT scans of the patient. (A) The initial CT scan demonstrates unclear low density in the left frontal, temporal and insular lobes and in the left thalamus. (B) Six months later, the CT scan shows the enlarged extent of the low density in the left frontal lobe, with lesions in the left temporal and insular lobes and in the left thalamus nearly unchanged. CT, computed tomography.

**Figure 2:**
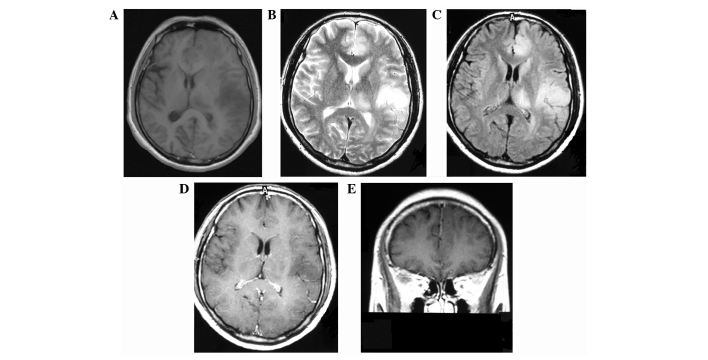
Axial MRI demonstrates (A) hypointensity on T1-weighted images and hyperintensity of (B) T2-weighted and fluid-attenuated inversion recovery images in the left frontal, temporal and insular lobes and in the left thalamus. (D) Axial T1-weighted MRI shows no enhancement within the lesion area, (E) while sagittal T1-weighted MRI shows no enhancement in the left frontal lobe lesion following gadolinium injection. MRI, magnetic resonance imaging.

The study was approved by the Ethics Committee of Liaoning Cancer Hospital & Institute (Shenyang, China). Written informed consent was obtained from the patient’s family. A 56-year-old man was admitted to the Department of Neurology, Dandong Central Hospital (Dandong, China) in June 2013 after 3 days of fever, dizziness, headache and numbness in the right extremities. Physical examination showed a blood pressure of 140/85 mmHg and a body temperature of 38.2°C. Neurological examination indicated mild memory deterioration and calculation impairment; however, reading and writing were normal. Examinations of motor, cerebellar function and gait were normal; however, the ability to sense pain and temperature in the right upper and lower extremities was impaired. On routine laboratory testing, the white blood cell count was observed to be increased to 15,200/mm^3^ (90.4% were neutrophilic leukocytes) and the C-reactive protein level was 1.0 mg/dl. The cerebrospinal fluid (CSF) obtained at admission showed mild pleocytosis of 10.0×10^6^/l leukocytes with 5% neutrophilic cells, 75% lymphocytes and 20% monocytes. The CSF protein concentration was 100 mg/dl, and the glucose level was 3.6 mmol/l. CSF gram stain, bacterial and viral cultures and HSV PCR were negative. Brain CT scans revealed abnormally low density in the left frontal, temporal, insular lobes and in the left thalamus (Fig. 1A). The results from the MRI demonstrated that all the lesions showed hypointensity on T1-weighted images (Fig. 2A), relatively homogeneous hyperintensity on T2-weighted images (Fig. 2B) and on fluid-attenuated inversion recovery (FLAIR) images (Fig. 2C). No signal-enhancement was observed following gadolinium administration (Fig. 2D and E). The patient received a 10-day course of acyclovir, intravenously. The patient was then discharged but the symptoms were not markedly relieved.

**Figure 3:**
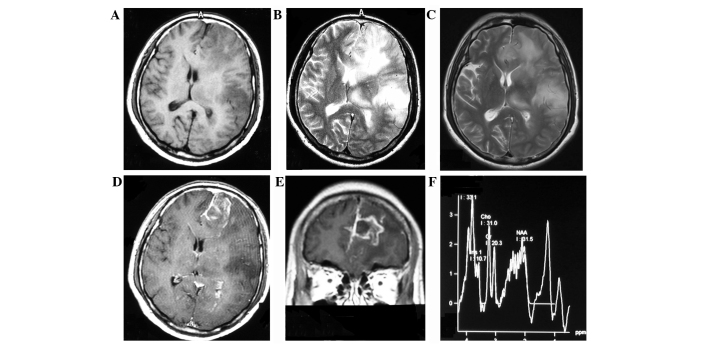
Axial MRI six months after presentation demonstrates the enlarged extent of the lesion in the left frontal lobe, with lesions observed in the left temporal and insular lobes and in the left thalamus nearly unchanged on (A) T1-weighted, (B) T2-weighted and (C) fluid-attenuated inversion recovery images. (D) Axial and (E) sagittal T1-weighted MRI shows irregular enhancement in the left frontal lobe lesion following gadolinium injection, with lesions in the left temporal and insular lobes and in the left thalamus nearly unchanged. (F) MRS shows elevated Cho/Cr, Cho/NAA ratios and decreased NAA/Cr ratios in the left frontal lobe lesion. MRI, magnetic resonance imaging; MRS, magnetic resonance spectroscopy; Cho, choline; Cr, creatine; NAA, *N*-acetylaspartate.

At the follow-up three months later, the patient presented with personality changes and memory deterioration. The follow-up MRI showed no remarkable changes. At the follow-up six months after presentation, the patient had expressive aphasia and severe headache. Subsequently, the patient had two tonic-clonic seizure onsets and was admitted to the Department of Neurosurgery, Liaoning Cancer Hospital & Institute (Shenyang, China). Physical examination was normal; however, neurological examination showed expressive aphasia, disorientation, memory deterioration, calculation impairment and strength weakness in the right extremities. The CSF was essentially normal. Brain CT scans revealed the enlarged extent of the low density in the left frontal lobe (Fig. 1B); however, the lesions in the left temporal and insular lobes and in the left thalamus were nearly unchanged. The MRI showed that the lesion had increased in size with more edema around the lesion in the left frontal lobe (Fig. 3A–C). Irregular enhancement signals were observed following gadolinium administration (Fig. 3D and E). The results from the magnetic resonance spectroscopy (MRS) showed elevated choline (Cho)/creatine (Cr) and Cho/*N*-acetylaspartate (NAA) ratios, as well as decreased NAA/Cr ratios in the lesions (Fig. 3F). Surgery was performed and the neuropathological diagnosis of WHO grade III astrocytoma was confirmed.

## Discussion

The term gliomatosis cerebri (GC) was first proposed in 1938 by Nevin (5). GC is a rare glial neoplasm, characterized by extensive diffuse brain infiltration and relative preservation of the underlying architecture. GC is an intriguing disease for several reasons. Firstly, it is difficult to distinguish between GC and diffuse gliomas. In this regard, GC may represent the most invasive form of diffuse gliomas. Secondly, in terms of histological grading and clinical course, GC is a heterogeneous disease, ranging from rapidly evolving to slowly and somewhat indolent forms. Due to the extensive spread of the disease, surgery, apart from biopsy for diagnosis, is rarely performed for patients with GC (6). Pathologists have described two types of primary GC. Type 1 is a classical form of GC, characterized by diffuse overgrowth, with neoplastic glial elements and without a focal mass presence. Type 2, which may stem from type 1, is characterized by a diffuse brain infiltration and focal mass presence, and is usually a high-grade glioma (7).

GC may occur at any age; however, the majority of patients are 40–50 years old. Clinical manifestations include headaches, seizures, visual disturbance, corticospinal tract deficit, lethargy and dementia (8). Correct diagnosis may be acquired in the majority of GC cases via clinical manifestations, neurologic examination, MRI scans and MRS. MRI reveals widespread invasive lesions with hypointensity on T1-weighted images and hyperintensity on T2-weighted images and FLAIR sequences. Furthermore, the abnormality is shown more clearly on FLAIR sequence images than on T2-weighted images. MRS usually shows elevated Cho/Cr and Cho/NAA ratios, as well as decreased NAA/Cr ratios in the lesions (9).

In general, the differential diagnosis of GC includes multiple sclerosis, viral encephalitis, adrenoleukodystrophy, metachromatic leukodystrophy and subacute sclerosing panencephalitis since the cerebral lesions are confluent and extensive on CT and MRI scans (3). Brain biopsy is used when differential diagnosis via clinical manifestations, CSF testing, MRI and MRS is very challenging. As a result of the diffuse infiltration of the neoplasm, surgery is not suitable. However, it may be used when the neoplasm grows into a large mass and high intracranial pressure is induced.

In the present study, a patient with type 2 GC mimicking acute viral encephalitis following clinical onset is presented. In particular, the malignant transformation of lesions into astrocytoma in the left frontal lobe was observed, while lesions in other areas were nearly unchanged after six months. This suggests that the neoplasm cells in the left frontal lobe may be different from those in other areas. All the neoplasm cells may be at distinct stages of malignant transformation; however, the cells in the left frontal lobe may be more likely to undergo malignant transformation than others. This confusing clinicoradiological profile led to the initial misdiagnosis of acute viral encephalitis. At the three-month follow-up, the patient had personality changes and memory deterioration. In spite of this, the MRI scan showed no remarkable changes. Therefore, a brain biopsy is recommended for the diagnosis of GC so that the correct measures may be taken for the treatment of this disease as early as possible.
